# Joint Content Placement and Storage Allocation Based on Federated Learning in F-RANs

**DOI:** 10.3390/s21010215

**Published:** 2020-12-31

**Authors:** Tuo Xiao, Taiping Cui, S. M. Riazul Islam, Qianbin Chen

**Affiliations:** 1 School of Communication and Information Engineering, Chongqing University of Posts and Telecommunications, Nan-An District, Chongqing 400065, China; S180131014@stu.cqupt.edu.cn; 2 Chongqing Key Labs of Mobile Communications, Chongqing 400065, China; chenqb@cqupt.edu.cn; 3 Department of Computer Science and Engineering, Sejong University, Seoul 05006, Korea; riaz@sejong.ac.kr

**Keywords:** fog radio access network, content placement, storage allocation, federated learning

## Abstract

With the rapid development of mobile communication and the sharp increase of smart mobile devices, wireless data traffic has experienced explosive growth in recent years, thus injecting tremendous traffic into the network. Fog Radio Access Network (F-RAN) is a promising wireless network architecture to accommodate the fast growing data traffic and improve the performance of network service. By deploying content caching in F-RAN, fast and repeatable data access can be achieved, which reduces network traffic and transmission latency. Due to the capacity limit of caches, it is essential to predict the popularity of the content and pre-cache them in edge nodes. In general, the classic prediction approaches require the gathering of users’ personal information at a central unit, giving rise to users’ privacy issues. In this paper, we propose an intelligent F-RANs framework based on federated learning (FL), which does not require gathering user data centrally on the server for training, so it can effectively ensure the privacy of users. In the work, federated learning is applied to user demand prediction, which can accurately predict the content popularity distribution in the network. In addition, to minimize the total traffic cost of the network in consideration of user content requests, we address the allocation of storage resources and content placement in the network as an integrated model and formulate it as an Integer Linear Programming (ILP) problem. Due to the high computational complexity of the ILP problem, two heuristic algorithms are designed to solve it. Simulation results show that the performance of our proposed algorithm is close to the optimal solution.

## 1. Introduction

Recently, the increasing popularity of intelligent devices such as wearable devices, smartphones and sensors in our daily life has triggered a surge in many distributed network devices, which results in massive amounts of heterogeneous data that need to be processed [1,2,3]. Due to such unprecedented amount of data with exponential growth trend [4], it becomes impractical to send all data to a remote cloud computing center for processing and is full of privacy issues [2]. In addition, some applications and services rely heavily on high-speed data rates and low latency transmission, which prompts the mobile network operators to rethink current network architectures and seek more complex and advanced technologies to bring content closer to end users with low latency and cost.

To satisfy the diverse multi-dimensional requirements of quality of service (QoS), such as low-latency transmission, enhanced broadband and ultra-reliability, a fog radio access network paradigm has been proposed as a promising evolution path for the future wireless network architecture [5,6]. By integrating fog computing into wireless networks, it enables the distribution of cloud computing power to the edge of the network, enabling context-aware services and applications to approach mobile users. With this location, fog devices provide a unique opportunity not only to implement edge caching, but also to perform edge processing. Therefore, we can intuitively use fog computing resources to design a new intelligent content caching and distribution mechanism, which are more flexible and can meet the QoS requirements of various application scenarios.

Due to the limited storage capacity of edge nodes, the network performance can be effectively improved by predicting the content popularity and actively caching the most popular one. However, most existing caching schemes are designed for highly controlled environments where users need to upload local private data to a central server, which may pose privacy and security risks [7]. Furthermore, with the increase of the number of users and data, the unreliability and communication cost of wireless networks cannot be ignored. Therefore, it is necessary to study a new network architecture with low communication cost and high reliability. To improve the caching performance of the edge network, a federated learning framework [1,2,8,9] is introduced to effectively predict the distribution of content popularity in the network. As a data-level distributed learning paradigm, federated learning is seen as a promising approach to generate high-quality models without having to collect all the local data at the server [10]. In the federated learning framework, each client trains its model based on the local data, and updates the global model accordingly by uploading the results of the training to the fog server. The fog server then returns the improved global parameters to user so that a new round of local training can begin. Finally, through model-level collaboration between the client and server, an accurate learning model can be generated. The core benefit of federated learning is to spread content over a large number of devices rather than having to centralize training data [11,12]. By applying federated learning to demand prediction problems, the users preference can be accurately predicted [13]. The realization of federated learning requires network edge devices to have powerful computing capabilities and flexible collaboration. Due to the sufficient fog computing resources, the F-RANs paradigm can fully support this.

The rapid increase in mobile data traffic places a heavy burden on the fronthaul link which connects fog servers to remote cloud centers. By caching content at a fog server, the content delivery rate can be improved, the cost of network traffic transmission can be reduced [14] and the quality of data transmission can be guaranteed. When a user requests content, the fog server that caches the content can provide the data directly, rather than fetching the content from a remote cloud computing center. Therefore, how to place the content on which caches nodes in the network is critical. Furthermore, caching performance is highly correlated with the capacity of storage. If the fog server is allocated with less storage, only a limited amount of content can be cached, which can result in a lower quality of service than larger cache storage. Therefore, to maximize the use of storage resources, an effective caching strategy must be designed to distribute storage across different network cache nodes, and storage resource allocation determines how many storages should be allocated to each fog server.

In this paper, we investigate jointly optimizing storage resource allocation and content placement in a caching enabled hierarchical F-RAN architecture, with the goal of minimizing network traffic costs. Moreover, considering users’ content requests and privacy security, we adopt a federated learning to make distributed prediction of user preferences in different F-APs and apply it to the design of cache policy. The proposed caching scheme can effectively improve the performance of network content caching.

The rest of this paper is organized as follows. We review related work in Section 2. The system model is described in Section 3. In Section 4, we describe the formulation of our optimization problem. A solution is provided in Section 5. Simulation results are discussed in Section 6, and the Conclusion and Future Work are drawn in Section 7.

## 2. Related Work

The core idea of F-RANs is to make full use of the rich computing resources, data sharing and storage capabilities of edge devices. Edge caching between Fog-computing Access points (F-APs) can bring content closer to mobile users, which effectively improves content delivery rate and reduces the heavy burden of link transmission.

Currently, some contributions focused on designing edge caching schemes or algorithms to improve the performance of F-RANs. The work of [15] summarized the latest progress in F-RANs performance analysis, which introduces advanced edge caching and adaptive model selection schemes to improve the spectrum and energy efficiency of F-RANs. Effective caching strategies for F-RAN was given in [16] where F-RAN refers to the cloud radio access network (C-RAN) architecture that utilizes distributed edge caching techniques [6,17]. The work of [18] discussed the impact of mobile social networks on the performance of F-RANs edge caching schemes from a perspective of users’ social relationships. The work of [19] make use of social information and edge computing to reduce the end-to-end delay effectively, and the network content caching, mobility management and wireless access control has been studied. Although some researches have been done on caching in F-RAN, there are few researches on the joint optimization of resource allocation and content placement in networks. In this paper, we focus on the optimization of joint content caching and resource allocation in the F-RAN architectures to further improve the caching performance of F-RANs.

Due to the limited storage capacity of edge devices, the content which is most likely to be requested by the user must be placed at the local fog server. The traditional cache mechanism updates the cache content based on static rules such as first in first out (FIFO) [20], least recently used (LRU) [21] and least frequently used (LFU) [22]. However, the popularity of content in the network changes over time, making this approach impractical. Currently, many researches focused on developing of dynamic caching schemes based on content popularity. The works of [23,24,25] modeled the caching problem as a Multi-Arm Bandit (MAB) problem, and indirectly obtained the content popularity distribution according to the cumulative request rate of all content. However, since the content popularity prediction process requires online cache training for all content, the prediction is not real-time and computationally complex. The work of [26] modeled the content popularity prediction problem as a Contextual Multi-Arm Bandit (CMAB) problem. In order to improve the accuracy of prediction, the scene information of all requesting users is partitioned, and the online prediction method similar to literature [23] is used in different scene partitions. Although the prediction accuracy has improved, it has not improved in terms of real-time prediction and computational complexity. The work of [27,28] used Alternating Direction Method of Multipliers (ADMM) algorithm to found highly popular content for caching through dynamic iteration. However, due to the prediction of content popularity in dynamic calculation, the cache timeliness cannot be guaranteed.

In addition, due to the dynamic nature of content and the mobility of user, the content popularity in the unit changes dynamically over time. Therefore, many studies use machine learning to learn content popularity by observing users’ historical content needs. Bastug et al. [29] proposed a small cellular network caching algorithm based on Collaborative Filtering (CF). However, CF algorithm has high computational complexity and is prone to cold-start when the data is sparse, which will affect the accuracy of content popularity prediction. The work of [30] proposed an active content caching mechanism based on transfer learning (TL), which was to minimize the content transmission cost of the system. It solves the problem of data sparseness, but if the similar content is migrated improperly, it will make the prediction accuracy worse. The work of [31] proposed using the Extreme Learning Machine (ELM) algorithm to build a model of the relationship between content features and user request information, and a random approximation algorithm is used for content feature selection design to improve the performance of the ELM algorithm. Finally, a trained model is used to predict future content popularity. However, the prediction algorithm cannot track the change of content popularity, and the algorithm accuracy still needs to be further improved.

Since most machine learning methods require the collection of individual user information at a central unit, which may cause privacy concerns for users. Local users have difficulty trusting the servers and are reluctant to upload their private data. In this context, federated learning as a distributed machine learning framework can effectively address this problem. It can perform the learning process from the data spread across multiple users, thus protecting sensitive data. Applying the federated learning framework to the demand predicting problem can effectively predict the distribution of network content popularity [32,33]. The performance comparison of content popularity prediction methods is provided in Table 1, and the main contributions of this paper are as follows:

- We jointly considered storage resource allocation and content placement in the network to formulate an optimization problem to minimize the network traffic cost.
- Due to the dynamic change of content popularity in the network, the federated learning framework is applied to predict the content popularity accurately in the region to develop an efficient content caching strategy. To the best of our knowledge, the problem of federated learning-based joint content placement and storage allocation has not been well studied in previous works.
- Two heuristic algorithms are proposed, and the experimental results based on real-worlds datasets verify the performance superiority of our proposed algorithm.

## 3. System Model

In this section, we first introduce a cache-enable F-RAN architecture and design a federated learning framework in F-RANs. Next, the content cache process is presented in details. Finally, the problem of content popularity prediction is formulated. Some key parameters are listed in Table 2.

### 3.1. System Architecture

**Figure 1:**
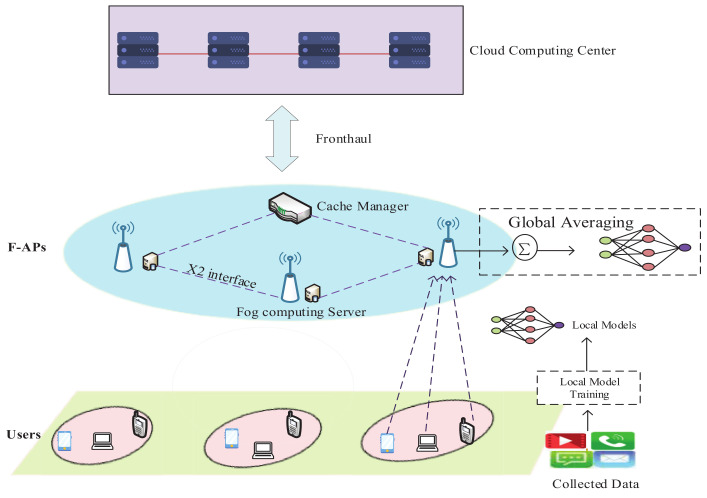
Federated learning paradigms in F-RANs.

An illustrative network architecture of F-RANs is shown in Figure 1. We consider a cache-enabled F-RAN architecture that contains *N* F-APs which is equipped with the fog computing server (denoted as a set N=1,2,…,N) and *U* mobile users (denoted as a set U=1,2,…,U ). F-APs exchange data with the cloud computing center through fronthaul links, and F-APs can communicate with each other and with a Cache Manager (CM) via X2 interface [5], to achieve content sharing. F-APs communicate with the users through wireless channels. We assume that each user can only download the requested content from the F-AP which is associated with it. Moreover, we consider allocating a certain amount of storage for each F-APs in the network, and the total caches of F-APs cannot exceed the upper limit *C* of the storage budget which is specified by the mobile network operator. CM can monitor all user-generated content requests [31] and is responsible for:

(1) Retrieve user’s requested content from the cloud computing server;
(2) Maintain an index table for storing cached content locations in the network;
(3) Forward the user’s content request to the neighboring F-APs that cache the content;
(4) Collect information about the requested content in F-APs;
(5) Decide when to update the entire content cache of F-APs, which can be refreshed at specific intervals or when content popularity changes significantly.

In the above-mentioned federated learning framework of F-RANs, each user can train a local model based on their own data, and then aggregate the local model at the fog computing server. The learning model is trained through the interaction between the fog computing server and the user until the model converges to a specific level of accuracy.

### 3.2. Caching Process

The mobile user connects a F-APs, and the connected F-AP is responsible for serving the user’s content requests. If a requested content is in the cache of the connected F-AP, the request is served immediately with no additional load placed on the fronthaul link, which reduces network traffic. On the other hand, if the F-AP does not cache the content requested by a local user, the request is forwarded to CM. The CM checks whether the contents requested in the lookup table are cached in neighbor F-APs. If the content is cached in the neighbor F-APs, CM will perform all necessary signaling to retrieve the content from the neighbor F-APs. Content provided by neighbor F-APs incurs lower downloading latency and reduces network traffic. Finally, if CM cannot find the requested content in any cache, it forwards the request to the remote cloud computing center for the content. Since dividing the content into small pieces and caching them at different levels will increase the complexity of the system, thus we assume that each piece of data is indivisible and can be cached on a F-AP as a whole.

Given the dynamic nature of network traffic, the content cached in the fog server should be updated regularly (e.g., an hour). At the beginning of each period, CM first optimizes content caching decisions and storage allocation strategies. If the reoptimization strategy is different from the previous phase, the cache can be updated and the cache storage can be reallocated accordingly.

### 3.3. Content Popularity

The set of popular content libraries requested by users in the network is represented as F={1,2,…,F}, and the average size of content is expressed as $s_{f}$. The status information from all users and each user requested content is defined as follows.

#### 3.3.1. Global Content Popularity

The global content popularity in the network is defined as $P_{f}$, which represents the probability distribution of content requested by all mobile users in the system. The popularity of the *f*-th content can be calculated as the ratio of the number of requests for *f* content to the number of requests for all content in the network. The common preferences of all users in the network can be expressed by the global content popularity, which usually follows a Zipf distribution model [34,35]:

(1) $$P_{f}=\frac{{\left(f\right)}^{-\beta }}{\sum_{j=1}^{F}{\left(j\right)}^{-\beta }}\;\;\;\;\;\;\;\;\;\;,\forall f\in F$$

where β is the skewness factor. The higher the value of β, the higher the number of requests concentrated on a few (popular) contents.

#### 3.3.2. Federated Learning Prediction

Due to different content preferences in different F-APs, and the probability that users from F-AP *n* requesting content *f* is defined as $P_{nf}$. User preferences can be predicted in advance or on a regular basis (e.g., hourly, daily, or weekly) through systematic learning and analysis of user social behavior [36,37]. In this paper, considering the privacy security of users, we adopt the federated learning method [7] to accurately predict the content popularity in the region.

**Figure 2:**
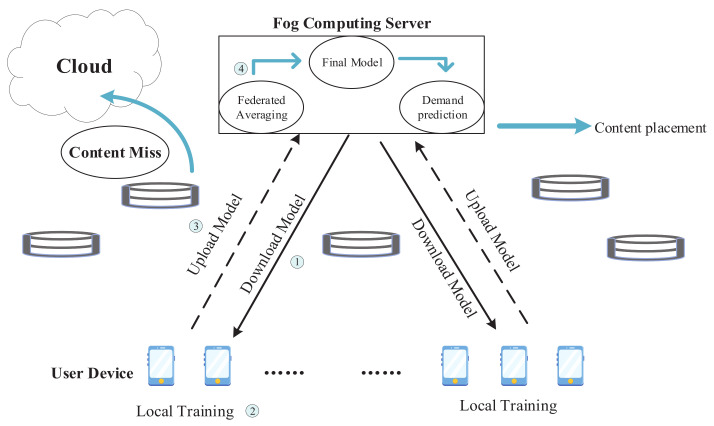
Federal learning prediction framework.

As shown in Figure 2, the federated learning framework includes the user’s device, which is responsible for local data training and uploading updates to the fog server. In general, the datasets used for local model training are generated based on the user’s device usage, such as the user’s web browsing and video playing in daily life. Different time and place, different activities, and even different types of mobile devices [26] may cause users to request different content. Therefore, the historical request information of users under different circumstances constitutes a part of the local training dataset. On the fog server, the global learning model is improved by merging and aggregating the local model updated from the user’s device. Finally, the fog server sends the improved model parameters back to the client, and this step is termed as a round of communication. The details of our designed FL communication process consist of the following steps:

① Model Download:

As shown in Figure 2, step ①, a set of users *U* are selected to participate in FL training for the *t*-th communication round. The selected users then download the global model from the fog computing server and train the model with their own local data. Therefore, they download the parameters $w_{u}$ of the global model from the fog computing server.

② Local Model Training:

The second step in our proposed FL is to train the model by utilizing local data at user devices, as shown in Figure 2, step ②. And at each round of algorithm iteration of *t*, the users participating in the training process are a subset of the entire user set. Each user *u* involved in the training process and updates its local parameter vector $w_{u}\left(t\right)$, implicitly built on the basis of its local dataset $\Delta_{u}$, in accordance with the following rule [2]:

(2) $$w_{u}\left(t\right)={\hat{w}}_{u}\left(t-1\right)-\alpha \nabla F_{u}\left({\hat{w}}_{u}\left(t-1\right)\right)$$

where α is the learning rate and ${\hat{w}}_{u}\left(t-1\right)$ represents the term $w_{u}\left(t-1\right)$ after global aggregation.

③ Upload Updated Model:

After completing the local model training, the users upload the local model parameters $w_{u}\left(t\right)$ to the fog computing server, as shown in Figure 2 step ③. In order to reduce communication costs and save the upload time, the model can be compressed before being uploaded to the fog computing server, as the uplink speed is slower than the download speed [38].

④ Weighted Aggregation:

After uploading their models, the last step is to generate the new global model w(t) by computing a weighted sum of all received local models $w_{u}\left(t\right)$, as shown in Figure 2, where *t* denotes the communication rounds in FL. The new constructed global model is used for the next training round. The fog server provides the weighted average suggested in [8], which is expressed as:

(3) $$w\left(t\right)=\frac{\sum_{u\in \{1,\ldots ,U\}}\left|\Delta_{u}\right|w_{u}}{\sum_{u\in \{1,\ldots ,U\}}\left|\Delta_{u}\right|}$$

where $|\Delta_{u}|$ indicates the cardinality of $\Delta_{u}$, i.e. the number of elements in $\Delta_{u}$.

The distributed data training of the algorithm proposed above has some advantages in terms of user privacy and content exchange. In fact, client is trained on local data, which allows users to protect their sensitive information. In addition, for each round of algorithm iteration, only a portion of the user set is involved, ensuring reduced messaging between the client and server. Finally, it should be emphasized that by considering the perspective of a user device, the gradient descent algorithm is used for optimization without excessive resource consumption. Therefore, after training a shared global model, each F-APs can predict local content popularity and then use it for cache content placement.

## 4. Problem Formulation

In this section, the problem is represented as a joint optimization of content caching and resource allocation with the objective of minimizing network traffic costs. The storage allocated for caching at F-AP *n* is denoted as $c_{n}$. A binary content cache matrix that is denoted as $X=\{x_{nf}|n\in N,f\in F\}$ indicates whether content *f* is placed at the local cache of server *n*. $x_{nf}=1$ if content *f* is precached in fog server *n* and $x_{nf}=0$ otherwise. The content popularity in the region is expressed as $P_{nf}$, that is, the probability of content *f* requested by the user of F-AP *n*, which can be predicted by using the federated learning method. Therefore, the problem is formulated as follows:

(4) $$\begin{array}{cc} \; & \min_{x,c}\sum_{f=1}^{F}\sum_{n=1}^{N}\{P_{nf}\cdot U_{n}\cdot s_{f}[W^{1}x_{nf}+\left(W^{1}+W^{2}\right)\left(1-x_{nf}\right)x_{mf}\\ \; & +\left(W^{1}+W^{3}\right)\left(1-x_{nf}\right)\left(1-x_{mf}\right)]\}\\ s.t.\; & C1:\sum_{n=1}^{N}c_{n}\leq C\\ \; & C2:\sum_{f=1}^{F}x_{n,f}s_{f}\leq c_{n},\;\forall n\in N\\ \; & C3:x_{nf}\in \left\{0,1\right\},\;\forall n\in N,\;\forall f\in F\\ \; & C4:x_{mf}\in \left\{0,1\right\},\;\forall m\in N,\;\forall f\in F \end{array}$$

where the first item of objective function (4) denotes that the content *f* is cached in the local F-AP *n* and constitutes the traffic through the wireless channel between user and F-APs. The second term indicates that content *f* is cached in neighbor F-APs *m* and constitutes the traffic through the Fog-Fog links and wireless channels. The third item represents that content *f* is requested from the cloud computing center and the traffic comes from the wireless channel and fronthaul links. The constraint C1 means that the cache allocated in all F-APs should not exceed the storage budget *C*. The constraint C2 means that all data in each F-APs should not surpass its storage capacities. The constraints C3 and C4 represent the caching decisions of fog servers in the network. Due to the product term in (4), the problem is nonlinear and difficult to solve. In this case, we introduce another binary decision variable $z_{nf}$ to enable $z_{nf}=x_{nf}x_{mf}$. In order to ensure that the transformed problem is equivalent to the original problem, the condition C5–C7 needs to be satisfied. Therefore, the converted problem can be expressed as follows: -4.6cm0cm

(5) $$\begin{array}{cc} \; & \min_{x,c,z}\sum_{f=1}^{F}\sum_{n=1}^{N}\{P_{nf}\cdot U_{n}\cdot s_{f}\left[\left(W^{1}+W^{3}\right)-W^{3}x_{nf}+\left(W^{2}-W^{3}\right)x_{mf}-\left(W^{2}-W^{3}\right)z_{nf}\right]\}\\ s.t.\; & C5:z_{nf}\leq x_{nf},\;\forall n\in N,\;\forall f\in F\\ \; & C6:z_{nf}\leq x_{mf},\;\forall m\in N,\;\forall f\in F\\ \; & C7:z_{nf}\geq x_{nf}+x_{mf}-1,\;\forall n,m\in N,\;\forall f\in F \end{array}$$

Transformation problem (5) is an integer linear programming (ILP) problem, which can be solved by exhaustive search algorithm, but with the high computational complexity and poor system performance. Therefore, two low complexity sub-optimal algorithms are designed to improve the performance of the system in the next section.

## 5. Problem Solution

Due to the high complexity of joint optimization problem calculation, in this section, we propose two sub-optimal heuristic algorithms to solve the problem, which can effectively improve the time efficiency. We decompose the joint optimization problem of storage allocation and content caching into two sub-problems. We first address the allocation of storage resources and then use it for the placement of cached content.

### 5.1. Storage Resource Allocation Problem

For storage allocation problems, storage resources are allocated to each F-APs based on the total F-APs storage budget. Therefore, the algorithm should be designed according to the different traffic requirements of F-APs to maximize the utilization of fog server storage resources. Traffic demand in the network is related to the popularity of the content, the number of users and the size of the content. For F-APs with high traffic requirements, more cache storage should be allocated. Therefore, we propose a traffic-based allocation algorithm, which allocates storage proportionally according to different traffic requirements. The Algorithm 1 in detail is shown as follows:
**Algorithm 1:** Traffic-based allocation Algorithm
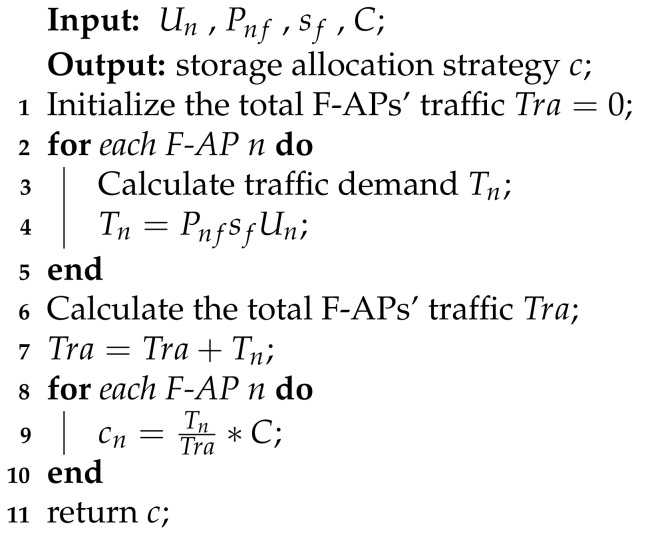


### 5.2. Cache Content Placement Problem

The content placement problem determines which content should be cached on each F-AP to minimize the traffic costs. Here two heuristics algorithms are proposed to address the problem of content placement.

#### 5.2.1. Greedy Algorithm based on Global Content Popularity

Due to the importance of content popularity in cache policy design, caching content with high popularity performs better. Greedy algorithm is adopted to cache as many popular content as possible on each cache entity. Specifically, the greedy algorithm based on global content popularity caches the most popular content in each F-APs until reaching the cache storage capacity limit. Algorithm 2 shows the process in detail. From the practical perspective, as different F-APs have their own preferences, the shortcoming of this algorithm is that it does not consider the content preference of regional users and the resource utilization is insufficient.
**Algorithm 2:** Greedy Algorithm based on Global Content Popularity
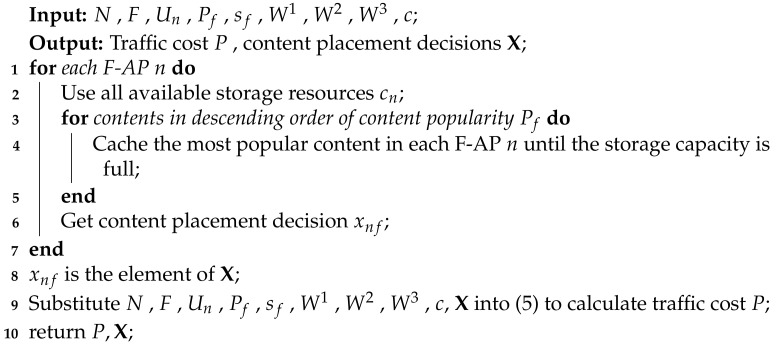


#### 5.2.2. Local Popularity Knapsack Algorithm based on Federated Learning

In this section, in order to reduce network traffic, a local popularity knapsack algorithm based on federated learning is proposed. The algorithm considers the local content popularity of each F-APs and avoids the deficiency of Algorithm 2. Firstly, the content that each fog server needs to cache depends on the content popularity $P_{nf}$ and the content size $s_{f}$. Therefore, the content caching decision of fog server *n* can be expressed as:

(6) $$\begin{array}{cc} \; & \max_{x}\sum_{f=1}^{F}P_{nf}s_{f}x_{nf}\\ s.t.\; & C8:\sum_{f=1}^{F}x_{nf}s_{f}-c_{n}\leq 0 \end{array}$$

where C8 means that all content cached in F-AP *n* should not exceed its capacity limit. According to Equation (6), we prefer to cache high popularity and large data length content in each fog server. It is observed that Equation (6) is a 0–1 knapsack problem [39], where $x_{nf}\in \left[0,1\right]$ is content placement decision, $x_{nf}=1$ means fog server *n* cache content *f*, otherwise $x_{nf}=0$, $s_{f}$ is the weight of content item *f*, $c_{n}$ is the knapsack capacity, and $P_{nf}s_{f}$ is the value of each item. Therefore, we can use dynamic programming [39] to solve the 0–1 knapsack problem.

The principle of dynamic programming is to divide the original problem into several subproblems and solve the subproblems by looking for the recurrence relation between the original problem and the subproblems, and finally achieve the effect of solving the original problem. In order to decompose the original problem into subproblems, a matrix *v* is constructed. If the knapsack capacity of the cached content item {1,2,…,f} is *j*, then v(f,j) represents the maximum target value that can be obtained. Therefore, the optimal solution is $v(F,c_{n})$, and the relationship between the original problem and the subproblem is:

(7) $$v\left(f,j\right)=\left\{\begin{array}{cc} v(f-1,j), & if\;j<s_{f}\\ max\{v\left(f-1,j\right),v\left(f-1,j-s_{f}\right)+P_{nf}s_{f}\}, & otherwise \end{array}\right.$$

If the storage capacity *j* is less than the size $s_{f}$ of the content item *f*, the fog server cannot cache the content. Therefore, we can remove content *f* in our solution and only consider caching data from {1,2,…,f−1}, that is v(f,j)=v(f−1,j). Otherwise, we choose the optimal one between caching or not caching the data for the content item *f*. The first item in curly bracket indicates that the content item *f* is not cached in F-APs and does not have any impact on the original problem or take up any storage. The second item means that the content item *f* is cached in F-AP, so the $P_{nf}s_{f}$ value is added to the original problem and occupies the storage capacity of $s_{f}$.

Algorithm 3 describes the knapsack algorithm process of local content popularity based on federated learning. With this algorithm, we can calculate all the entries of *v* and trace back the entries of *v* according to the optimal solution to determine the contents cached in F-APs *n*.
**Algorithm 3:** Local Popularity Knapsack Algorithm based on Federated Learning
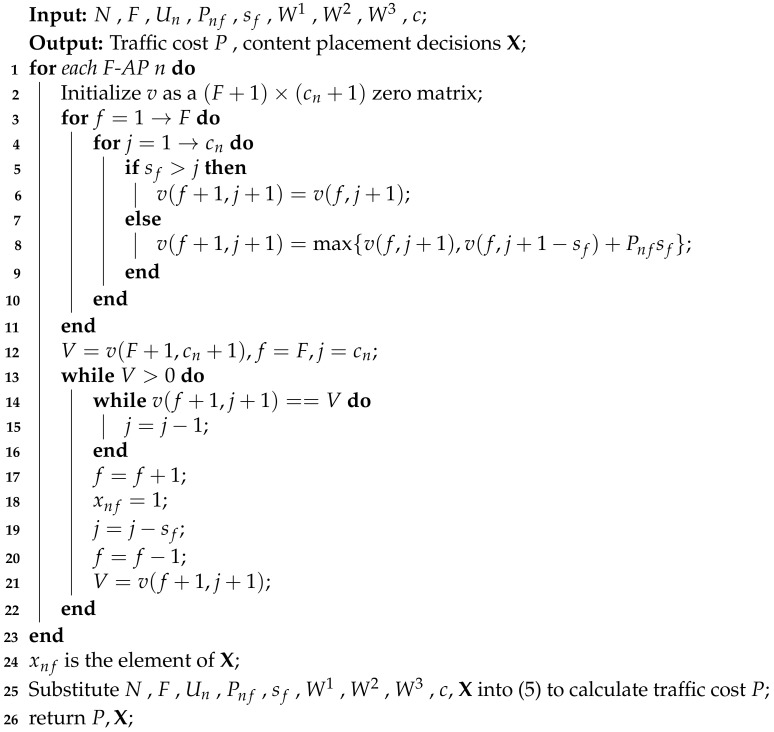


## 6. Simulation Results

In this section, the experimental results of the proposed algorithms are investigated, and the performance of three other algorithms, that is Oracle, No Storage Allocation and Random, are provided as references.

### 6.1. Simulation Parameters

In our simulation, we set the number of F-APs N=30. Since more users are sharing a fronthaul link than a Fog-Fog link in the network, the fronthaul link is more likely to cause traffic congestion. Therefore, the traffic cost of wireless link, Fog-Fog link and the fronthaul link are $W^{1}$, $W^{2}$ and $W^{3}$ respectively, where $W^{3}>W^{2}>W^{1}$. According to the parameters used in [40], in our simulation experiment, the values of $W^{1}$, $W^{2}$ and $W^{3}$ are set to 1, 2, and 4 per MB, respectively. Mobile users are randomly distributed among different F-APs, where the number of users U=1000. The average size of content is $s_{f}$, and the actual size of all content in the network is randomly selected from $0.8s_{f}$ to $1.2s_{f}$. That global content popularity in the network can be represented by Zipf distribution with β=0.56, which agrees with the used models in [34]. Since different F-APs have their own preferences, we represent the probability of user request *f* in F-AP *n* as $P_{nf}$. Considering the privacy security of users, federated learning framework is adopted to accurately predict content popularity in F-APs. The parameters used in the simulation experiment are shown in Table 3.

### 6.2. Datasets

In our experiment, we used real-world datasets—MovieLens [41]. The MovieLens dataset contains ratings data for multiple movies by multiple users, as well as movie metadata information and user attribute information. The MovieLens 1M dataset contained 1,000,209 ratings for 3706 movies participated by 6040 users, while the MovieLens 100K dataset had 100,000 ratings for 1682 movies from 943 users. Each user reviews at least 20 movies, and the user rating is based on a five-star scale, that is, from 0 to 5. In this paper, to simulate the process of users requesting content, we assume that the movie participating in the rating is the content requested by the user, and each movie rating corresponds to a content download. The paper of [26,42] adopt a similar method to simulate the process of users requesting content.

### 6.3. Evaluation and Discussion

The evaluation is based on two different sizes of datasets: MovieLens 1M and 100K. And the algorithms proposed in this paper are compared with the following three algorithms:

(i) Oracle: The algorithm has a priori knowledge of content popularity and provides optimal cache performance.
(ii) No storage allocation (NoStrgAlloc): The content popularity follows the Zipf distribution and does not consider the storage resource allocation of the fog computing server.
(iii) Random: The random algorithm randomly selects *F* content for caching, which provides the lowest caching performance.

**Figure 3:**
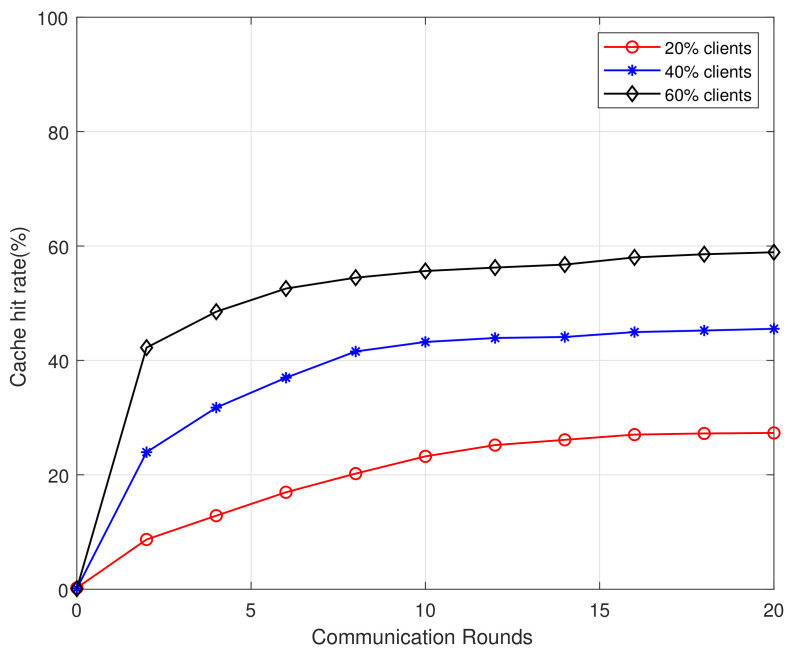
Cache hit rate vs communication rounds for datasts of MovieLens 100K.

**Figure 4:**
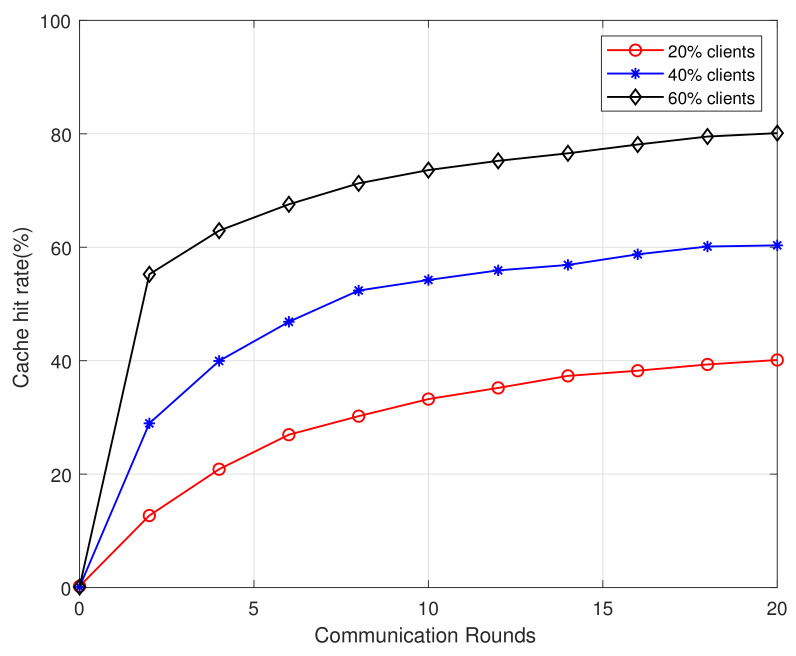
Cache hit rate vs communication rounds for datasts of MovieLens 1M.

Figure 3 and Figure 4 depict the cache hit rate (HR) against the number of federated communication rounds with different numbers of participated users. The results indicate that the more users participated in the learning process, which an accurate result can be trained to achieve better cache performance. In addition, fewer communication rounds are needed with more users or larger datasets. Therefore, the model can be updated by increasing algorithm rounds to improve system performance and make HR reach a higher value.

**Figure 5:**
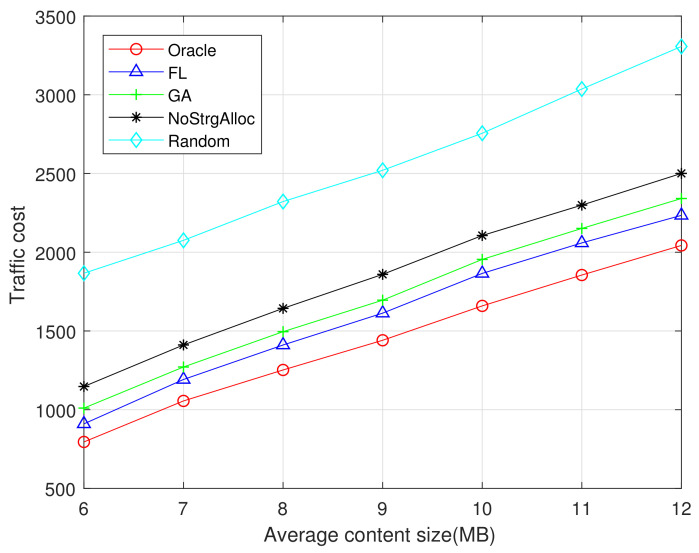
Traffic cost vs average content size (MovieLens 100K).

**Figure 6:**
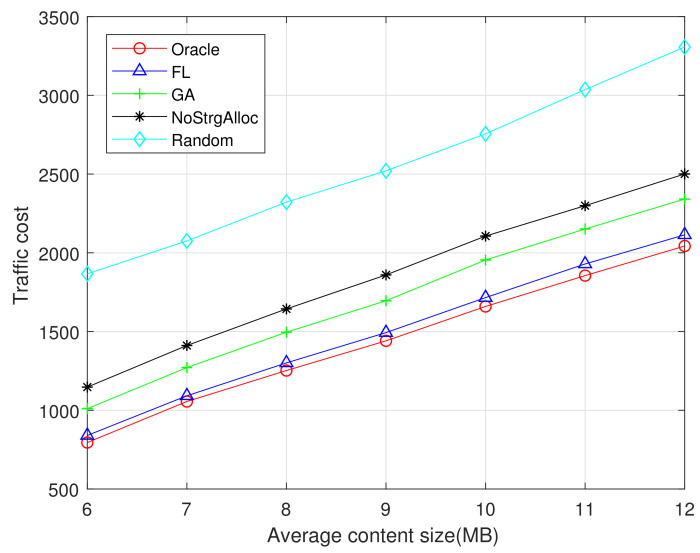
Traffic cost vs average content size (MovieLens 1M).

The performances of GA and FL algorithm under different average content sizes are evaluated. The total storage capacity of all F-APs in the network is set to C=1500 MB. Figure 5 and Figure 6 show the traffic cost with different average content sizes ranging from 6 MB to 12 MB. As can be seen from Figure 5 and Figure 6, the traffic cost increases with the average content size. Since the larger the content size is, the more network traffic will be generated. Oracle algorithms provide the lowest traffic cost because it has a perfect prior knowledge of future user requirements. The random algorithm has not considered the content popularity and the allocation of storage resources, thus resulting in the highest traffic cost. The algorithm without considering storage resource allocation in F-APs produces the second highest traffic cost. FL algorithm performs better than GA because FL considers local popularity instead of global popularity, avoiding the disadvantage we discussed earlier that only considered global popularity.

In comparing Figure 6 with Figure 5, observe that the performance of FL algorithm is closer to the Oracle (optimal) when the datasets is 1M rather than 100K, because FL algorithm has a better training effect when the datasets is larger and can predict the content popularity more accurately in the region. Due to the limited storage capacity of edge network nodes, pre-cached popular content can serve more user requests, thus reducing the network traffic costs effectively.

**Figure 7:**
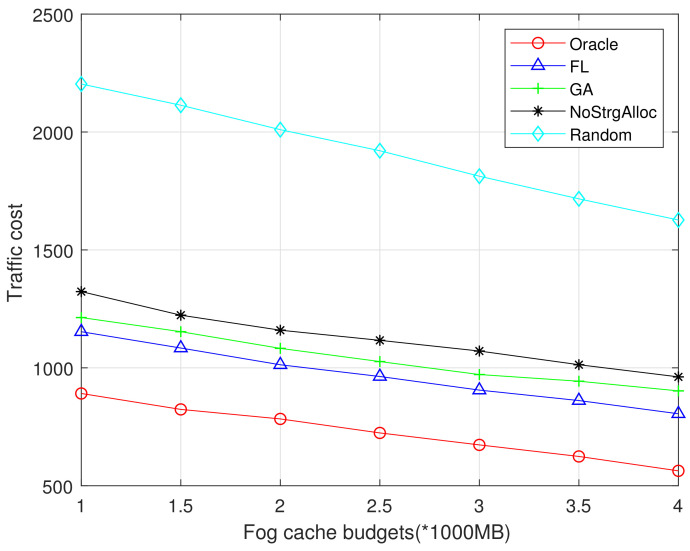
Traffic cost vs Fog cache budgets (MovieLens 100K).

**Figure 8:**
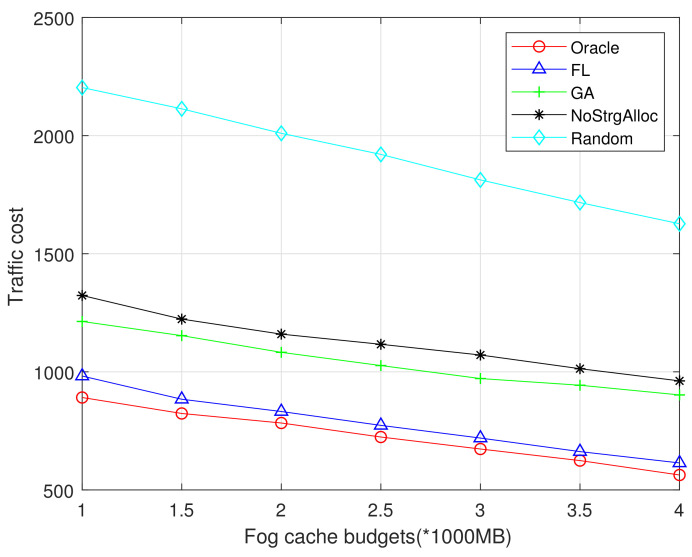
Traffic cost vs Fog cache budgets (MovieLens 1M).

The storage budget is an important metric that should be considered in designing caching strategies. Figure 7 and Figure 8 show the relationship between traffic costs and the F-APs cache budget. We compare the impact of F-AP cache budget *C* on traffic cost in different algorithms, and the average content size is set to 10 MB. In both Figure 7 and Figure 8, it is observed that the traffic cost decreases with F-APs cache budgets because more popular content can be cached in F-APs and hence less traffic is incurred to both the fronthaul and Fog-Fog links. The performance of Oracle algorithm is best, the random algorithm has the worst performance, and the cache without storage allocation is the second worst. FL performs better than GA because FL consider local popularity instead of global popularity. Compared with Figure 7, Figure 8 shows that the more datasets involved in model training, the better prediction can be achieved and the performance of the proposed algorithm is closer to the optimal one.

**Figure 9:**
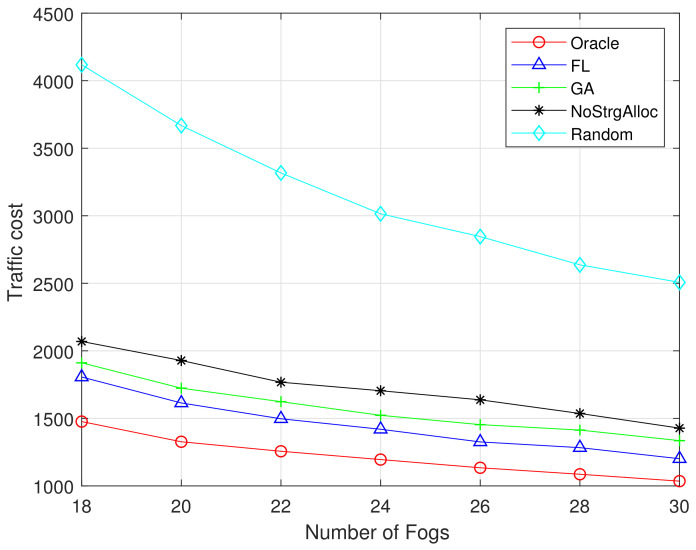
Traffic cost vs number of Fogs (MovieLens 100K).

**Figure 10:**
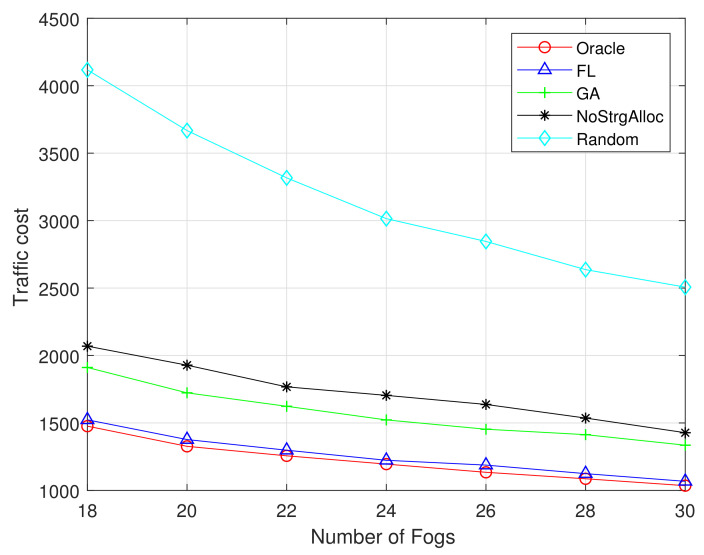
Traffic cost vs number of Fogs (MovieLens 1M).

Figure 9 and Figure 10 show the relationship between traffic costs and the number of F-APs. Both figures show that as the number of F-APs increases (from 18 to 30), the cost of traffic will decrease. Because the more F-APs there are, the more popular content can be cached in the local fog server, and the less traffic is injected into the network. The performance of our proposed FL and GA is superior to the existing random algorithm and no storage allocation algorithm. Moreover, the performance of FL algorithm is closer to the optimal one when the training dataset is 1M than that of 100K.

As is discussed above, the simulation results based on the real-world datasets show that the more users and datasets participate in the training, the better the prediction effect of content popularity. Therefore, it can better realize the allocation of storage resources and the popular content placement in the network, thus reducing the network traffic cost effectively.

## 7. Conclusions and Future Work

In this paper, a federated learning-based intelligent F-RANs cache architecture is investigated. In the F-RAN architecture, we presented joint optimization of content caching and resource allocation to minimizing the traffic costs under F-APs storage budget constraints. In addition, considering the user’s content request and privacy security, we adopt federated learning to make a distributed prediction of content popularity in different F-APs and apply it to the design of cache policy. The proposed caching scheme performs both efficient cache deployment and content caching. Due to the high computational complexity of the ILP model, and as the size of the problem increases, its scalability is not good. To reduce the computational complexity, two heuristic algorithms, that is greedy algorithm and FL algorithm, are introduced to provide approximate optimal solutions with lower computational complexity. Simulation results based on real-world datasets show that the proposed algorithm has better performance than existing algorithms and can obtain approximate optimal solutions.

Although the federated learning paradigm provides an efficient solution for implementing network edge smart caching in F-RANs, some key challenges remain. Due to the dynamic environment of network, mobile users may go offline or fall behind in the process of federated learning, which leads to poor accuracy of the training model. In future work, we will explore proactive content caching schemes based on fully asynchronous federated learning to better cope with highly dynamic network environments. 

## Figures and Tables

**Table 1:** Performance Comparison.

Related Work	[23,24,25]	[26]	[27,28]	[29]	[30]	[31]	This Work
Online/Offline-Learning	Online	Online	Online	Offline	Offline	Offline	Online
High Computational	Yes	Yes	Yes	Yes	No	Yes	No
Accuracy	No	Yes	Yes	No	No	No	Yes
Real-Time	No	No	No	No	No	No	Yes
Privacy Protection	No	No	No	No	No	No	Yes

**Table 2:** Key Parameters.

Notation	Definition
N	Set of F-APs
N	Number of F-APs
U	Set of mobile users
U	Number of mobile users
C	Storage budget of F-APs
F	Library of popular contents
F	Total number of contents
$s_{f}$	Size of content f
$p_{f}$	Global content popularity
β	The skewness factor of Zipf
$P_{nf}$	Local content popularity
$c_{n}$	The storage capacity of F-AP *n*
X	A binary content cache matrix
$x_{nf}$	Content cache decisions
$\Delta_{u}$	Local dataset
α	Learning rate
$w_{u}\left(t\right)$	Local parameter vector
$W^{1}$,$W^{2}$,$W^{3}$	The traffic cost of wireless link, Fog-Fog link and fronthaul link, respectively

**Table 3:** Simulation Parameters.

Parameter Name	Value
Number of F-APs	*N* = 30
Number of users	*U* = 1000
The traffic cost of wireless link	$W^{1}=1$ MB
The traffic cost of Fog-Fog link	$W^{2}=2$ MB
The traffic cost of fronthaul link	$W^{3}=4$ MB
The total storage budgets of F-APs	C=1500 MB
The average content size	$s_{f}=10$ MB
Zipf distribution skewness parameter	β=0.56

## Data Availability

Data sharing not applicable.
